# Cupping for psoriasis vulgaris: A protocol of systematic review and meta-analysis

**DOI:** 10.1097/MD.0000000000020348

**Published:** 2020-05-15

**Authors:** Jie Zhang, Qianying Yu, Li Peng, Feng Zhang, Wenxia Lin, Jing Guo, Min Xiao, Mingling Chen

**Affiliations:** Hospital of Chengdu University of Traditional Chinese Medicine, Chengdu, Sichuan, PR China.

**Keywords:** cupping, meta-analysis, protocol, psoriasis vulgaris, systematic review

## Abstract

**Background:**

Psoriasis vulgaris (PV) is a chronic, immune-mediated dermatological disease that significantly affects the patient's health and quality of life. At present, cupping has been widely used in the treatment of psoriasis. However, the effectiveness and safety of cupping in patients with PV are still controversial. Therefore, this review aims to evaluate the efficacy and safety of cupping therapy on PV.

**Methods:**

The following databases will be searched from their inceptions to April 2020 with a language limitation of English and Chinese: Pubmed, Medline, Embase, Cochrane Central Register of Controlled Trials, Chinese Biomedical Literature Databas, China National Knowledge Infrastructure Database, Wanfang database and Chinese Scientific Journal Database. The reference lists of eligible studies and other resources will also be searched. Two researchers will independently perform the selection of studies, data extraction, and data analysis. A fixed or random-effect model will be applied to synthesize data depend on the heterogeneity test. The primary outcome is the proportion of patients achieving at least a 60% improvement in psoriasis area and severity index (PASI) score from baseline (PASI 60). Secondary outcomes include the proportion of patients achieving at least a 90% improvement in PASI score from baseline (PASI 90), the mean change of PASI and dermatology life quality index score, the itching index, adverse events, and recurrence rate. RevMan V.5.3 software will be used for meta-analysis.

**Results:**

The study will provide a high-quality evidence-based review of cupping for PV.

**Conclusions:**

The study will be conducted to evaluate the efficacy and safety of cupping in the treatment of PV and supposed to provide clear evidence for the clinical application of cupping therapy.

**Ethics and dissemination:**

As the study is a protocol of systematic review and meta-analysis that does not involve individual data, ethical approval will not be required. The results will be published in a peer-reviewed journal.

**OSF Registration number:**

DOI 10.17605/OSF.IO/KV4CJ

## 1 Introduction

Psoriasis is a chronic, inflammatory skin disease with a global prevalence of 0.51% to 11.43% in adults and 0% to 1.37% in children.^[1]^ Psoriasis vulgaris (PV), the most common type of psoriasis, is characterized by erythematous plaques with silvery lamellar scales.^[2,3]^ The etiology and pathogenesis of psoriasis have not been fully elucidated. Yet, it has been recognized as a multifactorial disease, where interactions exist between genetic background, environmental factors, immunological factors, inflammation, and metabolism.^[4]^ Besides, season change, psychological stress, dietary factors, and alcohol consumption are the common factors contributing to the relapse or aggravation of the disease.^[3]^ As an immune-mediated systemic disease, psoriasis not only affect the skin but also affect the joints, and has been associated with different comorbidities, such as cardiovascular disease, metabolic syndrome, inflammatory bowel disease, hepatic disease, and so on.^[5]^ Some studies^[6,7,8]^ indicate that patients with psoriasis often suffer from anxiety, anger, depression, negative self-image, or difficulties in social interactions. Evidence also reveals that the economic burden of psoriasis including the direct and indirect costs is substantial and significant.^[9,10,11]^

According to German S3-guidelines on the treatment of PV,^[10]^ therapeutic approaches include topical therapy, conventional systemic therapy, phototherapy, and biologic. The topical treatments such as Corticosteroids, Vitamin D3 derivatives, and Tazarotene are recommended for mild psoriasis. Biologic agents are usually recommended for patients with moderate to severe psoriasis after failures of systemic therapy and phototherapy.^[12,13,14]^ Psoriasis is an incurable disease characterized by periods of recurrence and remission so that many patients require long-term care. However, due to the various side effects and high costs with long-term use of conventional systemic therapies or biologics, patient with psoriasis always report the low-level satisfaction with current therapies,^[15,16]^ and try to pursue the complementary and alternative medicine (CAM), which have not been considered as a part of conventional medicine.

Acupuncture is a substantial part of CAM, which is widely applied to treat psoriasis because of its confirmed efficacy, fewer side effects, and lower medical costs.^[17]^ As an important form of acupuncture, cupping therapy (CT) is an ancient method that has been used in different traditional medicine for thousands of years, especially in countries such as Egypt and China.^[18]^ CT is usually classified into 2 major types: dry or wet cupping, which has been used to treat various dermatological diseases including psoriasis, erysipelas, eczema, urticaria, acne vulgaris, herpes zoster, and associated post-herpetic neuralgia.^[19]^ Several studies indicate that CT is an effective measure to improve symptoms and reduce the recurrence rate of PV in China.^[20,21,22,23]^ However, the effectiveness and safety of CT on psoriasis are still controversial due to the low qualities of these studies and the cupping-induced koebner phenomenons reported in some cases.^[24,25]^ Therefore, it is necessary to conduct a high-quality systematic review based on the currently increased clinical trials about CT on PV. This review is performed to evaluate the efficacy and safety of CT on PV and supposed to provide clues for clinical application.

## 2 Methods

### 2.1 Study registration

The systematic protocol will be performed complying with the Cochrane Handbook and the Preferred Reporting Items for Systematic Reviews and Meta-Analyses Protocols statement guidelines.^[26]^ The OSF registration number is DOI 10.17605/OSF.IO/KV4CJ.

### 2.2 Eligibility criteria

#### 2.2.1 Types of studies

Randomized controlled trials (RCTs) will be included in our research. Other types of studies such as Quasi-RCTs, non-RCTs, case reports, case series, and animal mechanism studies will be excluded.

#### 2.2.2 Types of participant

Eligible patients must be diagnosed with PV according to at least 1 internationally or nationally authorized diagnostic criteria. Patients diagnosed with psoriatic arthritis, pustular psoriasis of palms and soles and erythrodermic psoriasis will be excluded. There will be no restrictions on age, gender, ethnicity, education status. The stage or severity of PV will not be limited.

#### 2.2.3 Types of interventions

##### 2.2.3.1 Experimental interventions

The experimental interventions should be either cupping alone or combined with another active treatment. Any cupping therapies (such as dry cupping, wet cupping, moving cupping, flash cupping, bleeding cupping, et al) will be considered. Combined cupping including at least 2 types of cupping therapies will also be considered. The type of cupping device will not be limited.

##### 2.2.3.2 Control interventions

The control interventions could be no treatment, placebo cupping, and another active treatment. The following comparisons will be considered if available:

(1) CT alone versus no treatment.
(2) CT alone versus placebo cupping.
(3) CT alone versus another active treatment.
(4) CT plus another active treatment versus the same active treatment.

#### 2.2.4 Types of outcome measures

##### 2.2.4.1 Primary outcome

Based on the psoriasis area and severity index (PASI) score decline rate, the proportion of patients achieving at least a 60% improvement in PASI score from baseline (PASI 60) is the primary outcome.

##### 2.2.4.2 Secondary outcomes

The secondary outcomes include: the proportion of patients achieving at least a 90% improvement in PASI score from baseline (PASI 90), the mean change of PASI and dermatology life quality index score compared with baseline, the itching index measured by the itching evaluation scale, adverse events, and recurrence rate during the follow-up period.

### 2.3 Search methods for the identification of studies

#### 2.3.1 Electronic searches

Pubmed, Medline, Embase, Cochrane Central Register of Controlled Trials, Chinese Biomedical Literature Database, China National Knowledge Infrastructure Database, Wanfang database and Chinese Scientific Journal Database will be searched from their inceptions to April 2020 with a language limitation of English and Chinese. The key search terms include psoriasis, psoriases, bai bi, CT, cupping, dry cupping, wet cupping, moving cupping, pricking cupping, bloodletting, pricking blood therapy. The search strategy for Pubmed is shown in Table 1 and modified by using other databases.

**Table 1:**
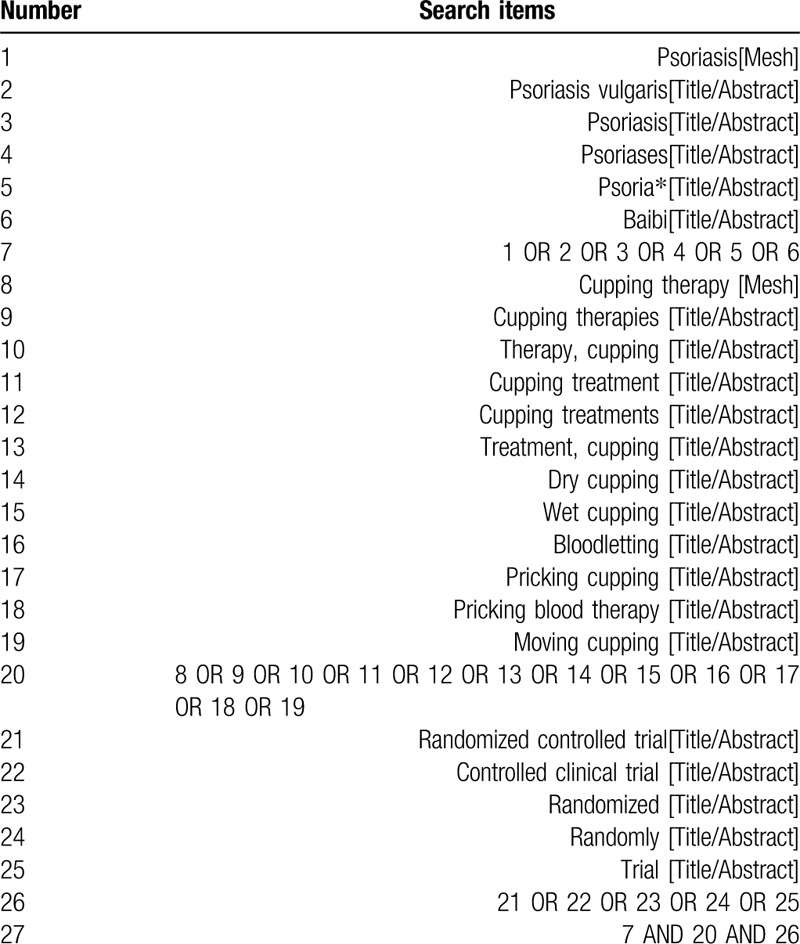
The search strategy used in PubMed.

#### 2.3.2 Other resources

The reference lists of relevant RCTs and review articles related to psoriasis treating with CT will be examined to identify potentially eligible studies. Published journals and conference papers related to the topic will be searched. Registers of clinical trials such as the ClinicalTrials.gov and the Chinese Clinical Trial Registry will also be searched.

### 2.4 Data collection and analysis

#### 2.4.1 Selection of studies

**Figure 1:**
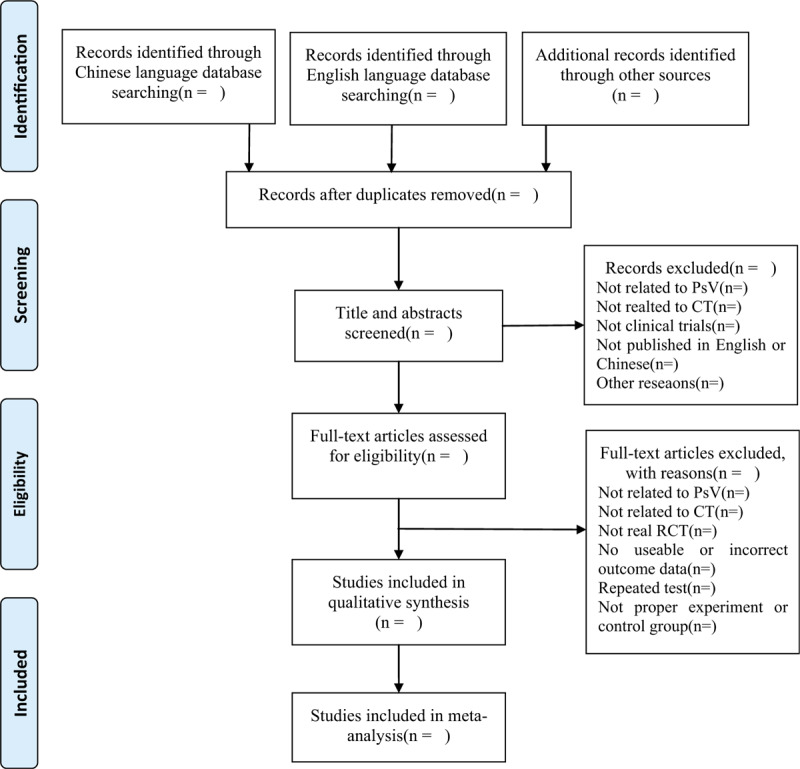
Flow diagram of the study selection process.

Before the selection of studies, a consensus on screening and subsequent procedures will be discussed among all researchers. The EndnoteX9 software will be applied to manage the searched literature. Duplicates will be excluded, then 2 qualified reviewers (JZ and QY) will independently screen the titles, abstracts, keywords, and full texts of the studies to select eligible trials. The reasons for excluded RCTs will be recorded in Excel data. Any disagreements during the study selection process will be resolved by the third investigator (LP). The flow chart of the study selection process is shown in Figure 1.

#### 2.4.2 Data extraction and management

Two reviewers (FZ and WL) will independently examine the eligibility of included studies and extract data. An extracted Excel data will include general information, study methods, participants, interventions and controls, outcomes, results, adverse events, conclusions. Any discrepancies and doubts will be resolved through discussion between the 2 authors (FZ and WL) or clarified with the third author (LP).

#### 2.4.3 Assessment of risk of bias in the included studies

The Risk of bias tool in Cochrane Collaboration's RevMan 5.3 software will be independently used by 2 authors (JG and MX) in the quality assessment. Seven domains of each trial will be assessed: random sequence generation, allocation concealment, blinding of participants and personnel, blinding of outcome assessment, incomplete outcome data, selective outcome reporting, and other bias. The study bias will be categorized into 3 levels: high risk, low risk, and unclear risk. Group discussions will be conducted to resolve any disagreements in consultation with the third author.

#### 2.4.4 Measures of treatment effect

For continuous data, the weighted mean difference or the standardized mean difference with 95% confidence intervals will be used to express the treatment effect. Risk ratio or odds ratio with 95% confidence intervals will be used to measure the treatment effect for dichotomous data.

#### 2.4.5 Dealing with missing data

To get missing or insufficient data of included studies, the first author or corresponding author will be contacted by email or telephone. If that fails, the existing data will be analyzed, and the potential impact of missing data will also be discussed.

#### 2.4.6 Assessment of heterogeneity

Statistical heterogeneity will be evaluated by the *I*^2^ statistic and chi-squared test. *I*^2^ < 50% will be considered as low or no heterogeneity, while *I*^2^ ≥50% will be considered to have significant heterogeneity among the trials. At that time, the sensitivity analysis or subgroup analysis will be conducted to explore the possible sources for heterogeneity.

#### 2.4.7 Assessment of reporting bias

If the number of included studies (more than 10 RCTs) is sufficient, funnel plots and Egger test will be used to evaluate publication bias.^[27]^

#### 2.4.8 Data synthesis

RevMan V.5.3 software will be applied for data synthesis. If the substantial heterogeneity is not detected (*I*^2^ < 50%), the fixed effects model will be used to perform a meta-analysis. The random-effects model will be applied to synthesize the data when the substantial heterogeneity is found (*I*^2^ ≥50%). The descriptive analysis will be conducted if the data cannot be synthesized due to the substantial heterogeneity.

#### 2.4.9 Subgroup analysis

If sufficient RCTs can be included, subgroup analysis will be carried out according to different types of cupping (dry cupping or wet cupping), intervention forms (cupping or cupping adjunctive to another therapy), and outcome measures.

#### 2.4.10 Sensitivity analysis

Based on sample size, methodological quality, missing data, and statistical models (fixed or random-effect model), sensitivity analysis will be conducted to identify the robustness and reliability of the results. The meta-analysis will be performed again after inferior quality studies have been excluded. The results of these meta-analyses will be compared and discussed according to the sample size, the strength of evidence and influence on pooled effective size. However, if all included studies have a high risk of bias, the sensitivity analysis will not be carried out.^[28]^

#### 2.4.11 Grading the quality of evidence

The quality of evidence will be evaluated using the grading of recommendations assessment, development, and evaluation profiler software (Version 3.6, The grading of recommendations assessment, development, and evaluation Working Group). The strength of evidence will be divided into 4 levels: high, medium, low, and extremely low.

## 3 Discussion

With the high prevalence, frequent recurrence, and the increased risk of comorbidities, psoriasis has become a serious public health problem, which significantly affects the patient's quality of life. Based on the various side effects and high medical costs with long-term use of current therapies that are recommended by clinical guidelines, it is not surprising that many patients report low levels of satisfaction with the treatment received and turn to apply CAM. In recent years, CT has been widely used in the treatment of psoriasis because of its convenience, and the low economic burden. At present, it is unclear whether CT is an effective and safe intervention for PV. Therefore, this review will evaluate current published studies to provide objective evidence of the efficacy and safety of CT for PV. However, the systematic review also has several potential limitations. Different types of CT, the severity and course of PV may contribute to the high heterogeneity. Besides, the language that studies published is limited to English or Chinese, which may result in selection bias.

## Author contributions

**Conceptualization:** Jie Zhang.

**Data curation:** Jie Zhang, Qianying Yu, Li Peng, Feng Zhang, Wenxia Lin.

**Formal analysis:** Jie Zhang, Qianying Yu, Li Peng.

**Funding acquisition:** Jing Guo, Mingling Chen.

**Investigation:** Wenxia Lin, Min Xiao.

**Methodology:** Jie Zhang, Qianying Yu, Li Peng, Feng Zhang.

**Software:** Feng Zhang

**Supervision:** Mingling Chen.

**Writing – original draft:** Jie Zhang.

**Writing – review and editing:** Mingling Chen, Jing Guo, Min Xiao.
